# AI‐Assisted Tumor Boundary Delineation via Targeted Ultrasmall Iron Oxide Nanoprobe for High‐Contrast HER2‐Positive Tumor Imaging

**DOI:** 10.1002/advs.77355

**Published:** 2026-08-24

**Authors:** Jiaying Zheng, Yi Zhu, Xinrui Li, Sunxian Dai, Xiangyuan Ma, Hongwei Lu, Zhongling Wang, Benqing Zhou

**Affiliations:** ^1^ Department of Biomedical Engineering College of Engineering Shantou University Shantou People's Republic of China; ^2^ Department of Radiology Shanghai General Hospital Shanghai Jiaotong University School of Medicine Shanghai People's Republic of China

**Keywords:** 3D nnU‐Net, deep learning, HER2‐positive tumor imaging, magnetic resonance imaging, ultrasmall iron oxide nanoparticles

## Abstract

Breast cancer continues to be a leading cause of cancer‐related mortality in women globally, where precise diagnosis and clear tumor demarcation are critical for effective treatment. Herein, we developed a strategic platform that combines a novel ultrasensitive magnetic resonance (MR) contrast agent with deep learning to significantly enhance the tumor‐to‐normal ratio (TNR). We designed ultrasmall iron oxide nanoparticles (USIO NPs) conjugated with trastuzumab (Tmab) for targeted MR imaging of HER2‐positive breast cancer. The USIO@Tmab nanoprobe demonstrated excellent HER2 specificity and pH‐responsive activation. The relaxivity of the nanoprobe shifted from a low T_1_‐weighted intensity (r_1_ = 1.43 mM^−^^1^s^−^^1^) under physiological conditions to an enhanced value (r_1_ = 4.07 mM^−^^1^s^−^^1^) in the acidic tumor microenvironment due to the detachment of Tmab protein. Additionally, we employed the 3D nnU‐Net deep learning framework as a post‐processing visualization aid to enhance tumor boundary detection via image fusion, rather than to amplify the underlying MRI signal. This approach yielded high segmentation accuracy, with an Intersection‐over‐Union (IoU) of 0.88 and a Dice coefficient of 0.93. This strategy provided an additional 2.59‐fold increase in TNR and enabled the reconstruction of three‐dimensional (3D) tumor models, offering clinicians an intuitive visualization of tumor structure for precise diagnosis and surgical guidance.

## Graphical abstract

A novel targeted and pH‐responsive MRI contrast agent was integrated with a 3D nnU‐Net deep learning framework to enable accurate delineation of tumor boundary morphology in HER2‐positive breast cancer imaging.

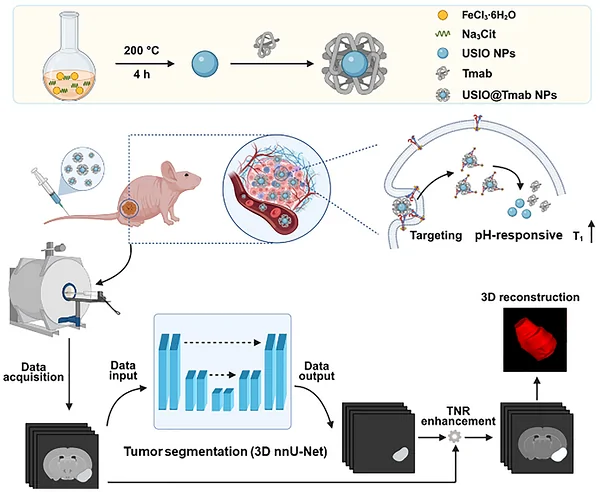

## 1 Introduction

Breast cancer is the most commonly diagnosed cancer and ranks as a major cause of cancer‐related deaths among women globally [1, 2, 3]. Among its subtypes, human epidermal growth factor receptor 2 (HER2)‐positive breast cancer accounts for approximately 20% of cases and is characterized by high invasiveness and metastatic potential, leading to an unfavorable prognosis [4, 5]. Precise diagnosis, including accurate delineation of tumor boundaries, is therefore a critical prerequisite for effective treatment [6]. Current clinical diagnosis of breast cancer primarily relies on imaging techniques such as ultrasonography and magnetic resonance imaging (MRI) [7].

MRI is recognized as one of the most powerful imaging techniques due to its high spatial resolution, non‐invasive nature, and strong ability to differentiate between soft tissues [8, 9, 10]. To achieve high diagnostic sensitivity, the administration of contrast agents is often necessary [11, 12, 13]. Currently, gadolinium (Gd)‐based T_1_‐weighted contrast agents are the clinical gold standard, however, they suffer from several limitations, including a short blood circulation half‐life that restricts the imaging window, nonspecific tissue distribution, and potential renal toxicity at high doses [14, 15]. As a promising alternative, ultrasmall iron oxide (USIO) nanoparticles (NPs) with diameters below 5 nm have emerged as attractive T_1_ contrast agents due to their superior biocompatibility and facile surface functionalization [16, 17, 18, 19]. For instance, zwitterionic L‐cysteine (Cys)‐coated USIO NPs (3.2 nm) have been engineered as an effective contrast agent, demonstrating antifouling properties and high r_1_ relaxivity for enhanced blood pool and tumor imaging [20]. To further enhance the diagnostic precision, we hypothesized that artificial intelligence (AI) algorithms could be utilized for postprocessing of MR images, thereby significantly increasing the tumor‐to‐normal ratio (TNR) in MRI and providing accurate and automated tumor boundary depictions [21, 22, 23, 24].

Recently, deep convolutional neural networks (CNNs), a prominent subset of AI algorithms, have driven substantial progress in medical image analysis, demonstrating remarkable performance in complex segmentation tasks [25, 26]. The application of CNNs in breast cancer diagnostics has a rich history, traceable to pioneering work in 1995 that utilized CNNs for classifying microcalcifications in radiographs of pathologic specimens [27]. Since then, the field has witnessed the development and adoption of increasingly sophisticated architectures—such as VGG‐16 [28], ResNet‐50 [29], Xception [30], and EfficientNet [31]—for the diagnosis and classification of breast cancer, significantly improving the efficiency and accuracy of early detection. Among these advanced frameworks, no‐new‐Net (nnU‐Net) has emerged as a powerful, self‐configuring, and open‐source tool specifically engineered for biomedical image segmentation [32, 33]. A key innovation of nnU‐Net is its ability to fully automate the segmentation pipeline, which includes dataset configuration, preprocessing, network architecture adaptation, training, and post‐processing, thereby minimizing the need for manual intervention and expert tuning [32]. It has set a new benchmark in semantic segmentation challenges, acclaimed for its robust generalization capabilities and streamlined workflow. Concurrently, tumor segmentation methodologies have evolved from manual delineation to semi‐automated and to fully automated approaches, with deep learning‐based methods becoming increasingly predominant [34, 35, 36, 37, 38]. These technological strides are paving the way for more accurate, efficient, and reproducible tumor diagnostics, offering particular promise in the context of breast cancer [39, 40, 41].

Recently, integrated strategies combining targeted delivery with pH‐responsive release have been extensively developed for enhanced tumor diagnosis and therapy [42, 43]. In this study, we developed a targeted and pH‐responsive nanoprobe by conjugating USIO NPs with trastuzumab (Tmab), a humanized monoclonal antibody that specifically binds to the HER2 receptor overexpressed on cancer cells [44, 45, 46, 47, 48]. The formed USIO@Tmab NPs exhibited excellent tumor specificity and biocompatibility. This study demonstrates that the combination of the USIO@Tmab nanoprobe with an AI‐based deep learning framework significantly enhanced TNR in MRI (Scheme 1). The USIO@Tmab nanoprobe is pH‐activatable, that is, it remains in a quiescent state as a T_1_ contrast precursor in normal tissues but is activated in the acidic tumor microenvironment (TME). This activation, through the release of USIO NPs, leads to a significant boost in the T_1_‐weighted signal specifically within tumors. Furthermore, when the acquired images were processed using the 3D nnU‐Net framework, a high segmentation accuracy was achieved, with a Dice coefficient exceeding 0.9, approximately 0.1 higher than that obtained without contrast agent administration. This AI‐powered enhancement provided an additional 2.59‐fold increase in TNR. Ultimately, this integrated technology enables the reconstruction of segmented two‐dimensional (2D) slices into detailed three‐dimensional (3D) tumor models, which precisely delineate tumor boundaries and volume. Most existing reports on AI‐ and deep learning–based segmentation have focused primarily on processing pre‐acquired medical images [49, 50]. The novelty of this work lies in the synergistic integration of a newly developed ultrasensitive contrast agent with an AI‐driven image analysis framework. Specifically, we propose a pH‐responsive and HER2‐targeted T_1_‐weighted nanoprobe (USIO@Tmab NPs) in conjunction with an advanced deep learning framework (3D nnU‐Net) to enable ultrasensitive mapping of tumor boundaries. This integrated approach provides a robust platform for precise diagnosis and intraoperative surgical guidance in HER2‐positive breast cancer.

**SCHEME 1:**
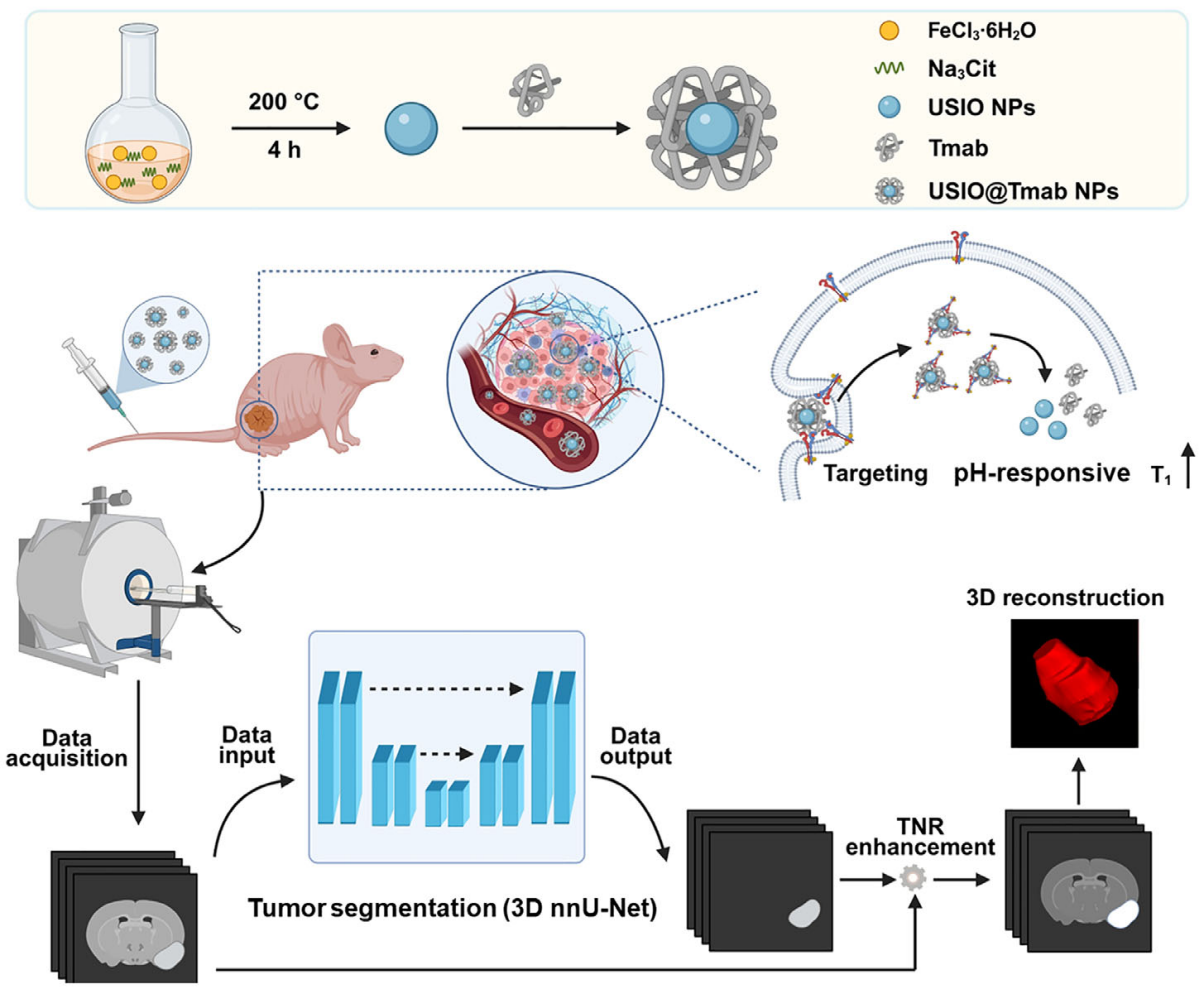
Schematic illustration of the synthesis of USIO@Tmab nanoprobe and the work mechanism of 3D nnU‐Net framework combined with MR images. The illustration was created with BioRender and used under license.

## 2 Results and Discussion

### 2.1 Synthesis and Characterization of USIO@Tmab NPs

The USIO NPs were synthesized via a solvothermal method. First, its morphological characterization by transmission electron microscopy (TEM) confirmed the formation of spherical nanoparticles with a narrow size distribution of 2.2 ± 0.6 nm (Figure S1a,b), while DLS revealed a hydrodynamic diameter of 9.8 nm (Figure S1c). Subsequently, the crystalline structure was confirmed by x‐ray diffraction (XRD), where the diffraction peaks at 30.2°, 35.3°, 43.1°, 53.3°, 57.1°, and 62.4° could be indexed to the (220), (311), (400), (422), (511), and (440) planes of the Fe_3_O_4_ crystal phase, respectively (JCPDS card No. 89–0688; Figure S2a) [51]. This crystal structure was further corroborated by selected area electron diffraction (SAED), which displayed a clear lattice pattern consistent with the XRD results (Figure S2b). Finally, x‐ray photoelectron spectroscopy (XPS) analysis was utilized to determine the surface composition (Figure S3). The characteristic binding energies observed at 710.8 eV for Fe 2p_3/2_ and 724.6 eV for Fe 2p_1/2_ confirm the formation of Fe_3_O_4_, thereby ruling out the presence of γ‐Fe_2_O_3_, in agreement with the previous report [52].

Following conjugation with trastuzumab (Tmab), the USIO NPs exhibited an increase in size, with the core diameter measured by TEM rising to 3.2 ± 1.5 nm and the hydrodynamic diameter by dynamic light scattering (DLS) increasing to 77.9 nm (Figure S4). For TEM, the measured diameter increases because the conjugated Tmab protein layer, though partially electron‐transparent, contributes to the apparent particle boundary in the dried state. For DLS, the increase is more pronounced, as DLS detects the hydrodynamic diameter, which includes the extended hydration layer and the flexible antibody arms projecting outward in aqueous solution. Notably, the discrepancy between TEM and DLS size for protein‐coated nanoparticles reflects the hydrated vs. dried state, rather than particle aggregation. Additionally, the coating of Tmab did not significantly alter the shape and dispersibility of the USIO NPs (Figure 1a). High‐resolution TEM imaging confirmed the high crystallinity of USIO@Tmab NPs, as evidenced by clearly observable lattice fringes (inset of Figure 1a). Zeta potential measurements provided further evidence of successful surface modification (Figure 1b). The citrate‐stabilized USIO NPs possessed a negative surface charge of ‐29.5 ± 2.9 mV, which was reversed to a positive value of 13.8 ± 2.1 mV for USIO@Tmab NPs, attributable to the introduction of the positively charged Tmab (28.0 ± 0.7 mV). Quantitative analysis using a bicinchoninic acid (BCA) protein assay determined the Tmab loading efficiency to be 80.5%, corresponding to a mass ratio of Tmab to USIO NPs of 0.58:1, as calibrated against a BSA standard curve (Figure S5). Fourier transform infrared (FTIR) spectroscopy further corroborated the successful conjugation (Figure 1c). Both USIO and USIO@Tmab NPs displayed the characteristic Fe–O stretching vibration at 586 cm^−^^1^, alongside citrate‐derived peaks at 1617 cm^−^^1^ (C═O), and 3415 cm^−^^1^ (O–H). It is notable that the peak of USIO@Tmab was significantly wider than that of USIO between 3300 and 3500 cm^−1^, demonstrating the presence of ‐N‐H in Tmab. Collectively, these results verify the successful preparation of the USIO@Tmab nanoprobe.

**FIGURE 1:**
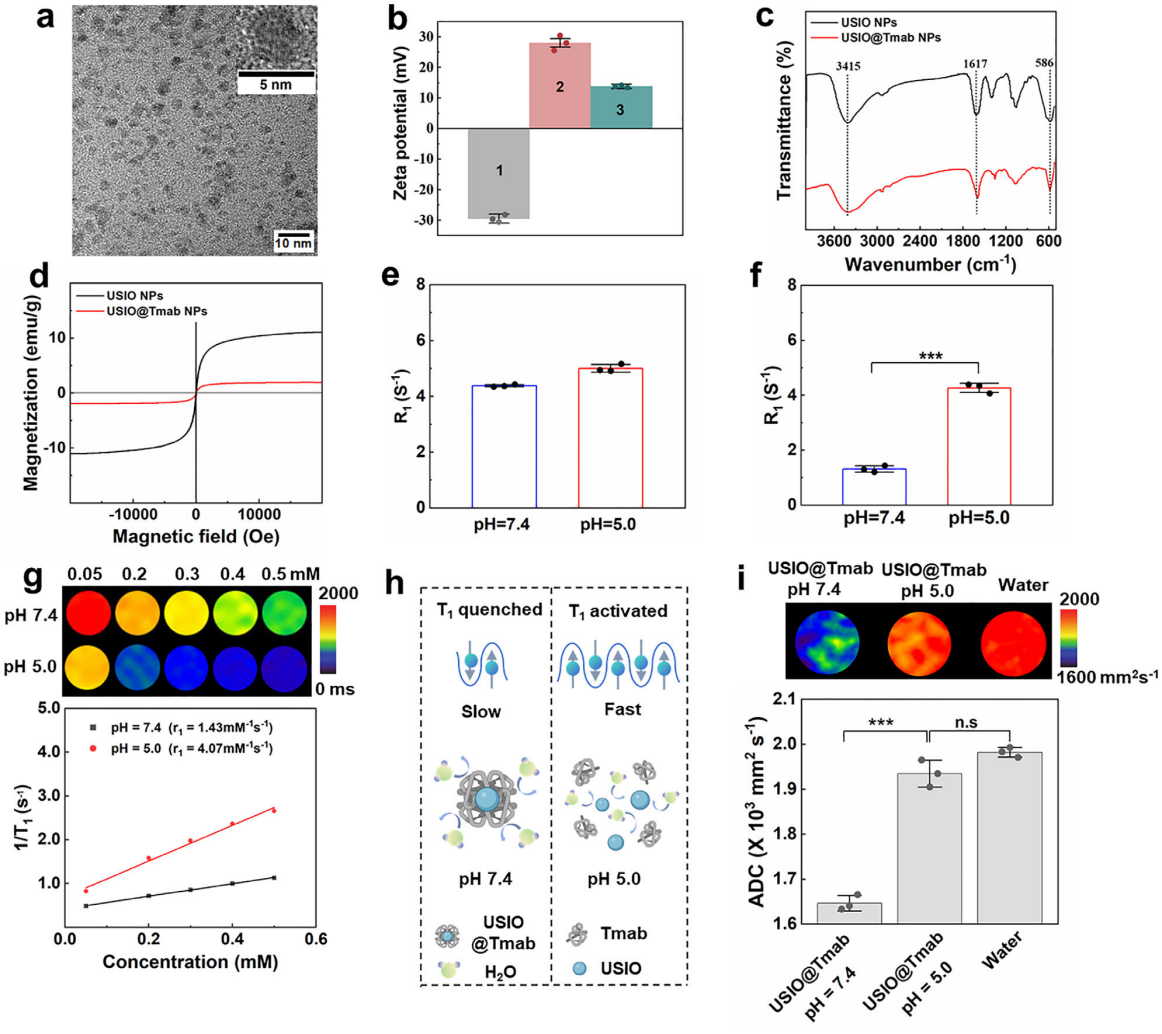
(a) A TEM image of USIO@Tmab NPs. The inset is the high‐resolution TEM image of a USIO@Tmab NP. (b) Zeta potentials of citrate‐stabilized USIO NPs (1), Tmab (2), and USIO@Tmab NPs (3), respectively. (c) Fourier Transform infrared spectroscopy (FTIR) spectra of USIO and USIO@Tmab NPs. (d) Magnetization hysteresis loops of USIO and USIO@Tmab NPs. (e, f) R_1_ of USIO NPs (e) USIO@Tmab NPs (f) at different pH conditions. (g) T_1_‐weighted color‐coded images and linear fitting of 1/T_1_ as a function of Fe concentration of USIO@Tmab NPs at different Fe concentrations. (h) The T_1_ activation mechanism of USIO@Tmab NPs under acidic conditions. (i) T_1_‐weighted images and corresponding ADC values of USIO@Tmab NPs at different pH conditions. Data are presented as mean ± SD (*n* = 3). n.s, not significant, and ^***^ *p* < 0.001.

The magnetic properties of USIO and USIO@Tmab NPs were investigated through vibrating sample magnetometry (VSM) at room temperature (Figure 1d). Both samples exhibited typical superparamagnetic behavior, characterized by the absence of coercivity and remanent magnetization (Mr), with magnetization returning to zero upon removal of the external magnetic field. The saturation magnetization (Ms) of the USIO NPs was measured to be 11 emu g^−^^1^, which is approximately consistent with the value reported in the literature [53]. After surface coating with Tmab protein, a reduction in saturation magnetization was observed for USIO@Tmab NPs, which is attributed to the introduction of the non‐magnetic protein layer coating the magnetic core.

It is worth noting that USIO and USIO@Tmab NPs both exhibited remarkable stability in water. Their hydrodynamic diameters remained unchanged over time in aqueous suspension, with no observable aggregation (Figure S6). Furthermore, the NPs maintained stability without any precipitation for 48 h in various physiological media, including water, phosphate‐buffered saline (PBS), and cell culture medium (inset in Figure S6), underscoring their excellent suitability for subsequent biological applications.

### 2.2 MRI Relaxivity and pH‐Responsive Mechanism of USIO@Tmab NPs

We next evaluated the T_1_ (T_1_ = Longitudinal relaxation time) relaxometry of the USIO@Tmab NPs. As shown in Figure 1e, the longitudinal relaxivity (r_1_) of USIO NPs does not change significantly at different pH levels (4.35 and 4.91 mM^−1^s^−1^), only increased by 12.9% after acid response. The r_1_ of USIO@Tmab NPs was relatively low at neutral pH (1.43 mM^−1^s^−1^); however, their value soared to 4.07 mM^−1^s^−1^ under acidic conditions (pH 5.0), their relaxation rate increased by 284.6% after acid response, and recovered 80% r_1_ of USIO NPs (Figure 1f). In addition, the MR signal intensities of USIO@Tmab NPs and USIO NPs are both Fe concentration‐dependent, and a higher Fe concentration leads to a higher MR signal intensity (Figure 1g and Figure S7). By linear fitting the inverse T_1_ as a function of Fe concentration, we can calculate the 1/T_1_ relativities. These results highlight the remarkable potential of the USIO@Tmab NPs nanoplatform to respond to alterations in the TME.

We next investigated the underlying mechanism behind the acidic pH‐triggered enhancement of T_1_ relaxivity (Figure 1h). Under acidic pH conditions, the USIO@Tmab nanostructure undergoes disintegration, thereby releasing the encapsulated USIO particles. The release accelerates longitudinal magnetization recovery and amplifies spin fluctuations by enhancing proton exchange between USIO and surrounding water molecules. Consequently, the T_1_ relaxation process is accelerated, resulting in a reduced T_1_ relaxation time. Accordingly, the pH‐responsive USIO@Tmab nanoprobe exerts a significant influence on T_1_ relaxation time by modulating spin fluctuations and local magnetic field variations, thereby generating brighter T_1_‐weighted images. To further explore this mechanism, we employed diffusion‐weighted imaging (DWI) to determine whether the pH‐responsive disassembly of USIO@Tmab NPs facilitates the diffusion of water molecules in the vicinity of the nanoparticles. ADC mapping revealed significantly lower diffusion coefficients for the USIO@Tmab NPs at pH 7.4 compared to pH 5.0 (Figure 1i). Therefore, we conclude that the activation effect of the T_1_ signal may be attributed to its acidic responsiveness, which promotes the diffusion of water surrounding magnetic ions. We therefore attribute the T_1_ signal enhancement to the pH‐responsive behavior that facilitates water diffusion in the vicinity of the magnetic ions.

### 2.3 In Vitro Cytotoxicity and Targeting

Prior to the use of the USIO@Tmab NPs for in vivo MRI applications, it is necessary to evaluate their cytocompatibility in vitro. The cytotoxicity of the USIO@Tmab NPs was investigated by CCK‐8 assay. The viabilities of 4T1‐HER2 and 4T1 cells were both higher than 90% even at the iron concentration up to 100 µg/mL (Figure 2a). The morphology of these cells was further observed under fluorescence microscopy (Figure S8). The cells co‐cultured with USIO@Tmab NPs showed no apparent morphological changes compared with the cells treated with PBS, as the cytoskeleton remained intact and the cytoplasm remained basically unchanged. These results suggest that USIO@Tmab NPs display negligible cytotoxicity in the given concentration range.

**FIGURE 2:**
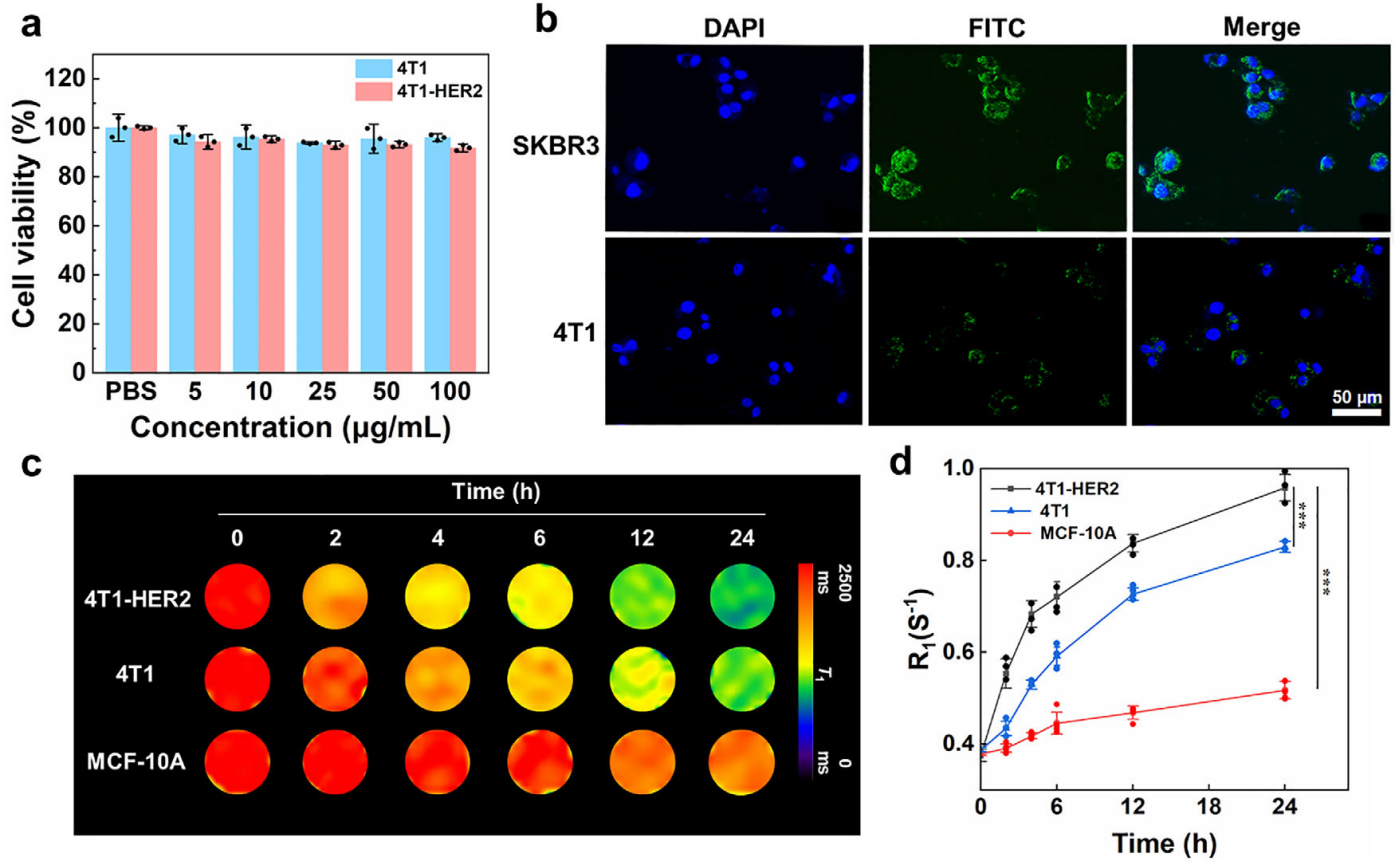
(a) CCK‐8 assay was utilized to analyze the cell viabilities of 4T1 and 4T1‐HER2 cocultured with USIO@Tmab NPs at different iron concentrations. (b) Fluorescence images of SKBR3 and 4T1 cells treated with FITC‐labelled USIO@Tmab NPs. The cell nucleus was stained with DAPI. (c, d) Representative T_1_ mapping images (c) and the corresponding graph of R_1_ values (d) of 4T1‐HER2, 4T1, and MCF‐10A cells cocultured with USIO@Tmab NPs at the iron concentration of 50 µg/mL, and collected at different time intervals (0, 2, 4, 6, 12, and 24 h). Data are presented as mean ± SD (*n* = 3), and ^***^ *p* < 0.001.

To explore the in vitro targeting specificity of USIO@Tmab NPs, SKBR3 (a HER2‐overexpressing cell line) and 4T1 (a HER2‐negative cell line) cells were incubated with FITC‐labelled USIO@Tmab NPs. As shown in Figure 2b, a strong fluorescent signal was localized predominantly on the membrane of SKBR3 cells, whereas no significant fluorescence was detected on or in 4T1 cells. This result demonstrates the superior targeting specificity of USIO@Tmab NPs towards cancer cells overexpressing HER2 receptors.

### 2.4 In Vitro MR Properties

We next evaluated the time‐dependent and pH‐responsive MR signal activation in vitro. USIO@Tmab NPs were incubated with 4T1, 4T1‐HER2, and normal MCF‐10A cells for up to 24 h. Signal activation was measured at designated time intervals. Notably, unlike healthy cells, cancer cells rely on glycolysis, which leads to the accumulation of an acidic TME, even under aerobic conditions. In contrast to the cancer cell lines, no significant MR signal enhancement was observed in the normal MCF‐10A cells, as expected. Conversely, 4T1‐HER2 breast cancer cells exhibited a marked increase in R_1_ values over time. Prolonged incubation further amplified these differences, with T_1_ mapping images of 4T1‐HER2 cells showing progressively “activated” signals over time, highlighting the pivotal role of the acidic TME in MR signal activation (Figure 2c). Specifically, R_1_ values in the 4T1‐HER2 group steadily increased between 2 and 6 h after exposure to USIO@Tmab NPs, ultimately reaching 0.97 s^−1^ after 24 h, a 5‐fold increase relative to the MCF‐10A group (Figure 2d). This increase was attributed to enhanced lactate accumulation in 4T1‐HER2 cells. Although 4T1 cells lack HER2 expression, their R_1_ values also gradually increased over time, likely due to the acidic tumor‐like microenvironment facilitating partial T_1_ signal activation. Nevertheless, the R_1_ values of 4T1‐HER2 cells remained consistently higher than those of 4T1 cells at all examined time points (Figure 2d), indicating that HER2‐mediated recognition promotes preferential nanoprobe uptake and enhances MR signal activation.

### 2.5 In Vivo MRI of Tumors

To evaluate the in vivo acidic microenvironment‐responsive properties of USIO@Tmab NPs, 4T1 tumor‐bearing mice were intravenously administered the USIO@Tmab NPs and commercial Gd‐DTPA as controls. Nanoparticle accumulation at the tumor site was monitored using a 3.0 T MRI scanner, with T_1_‐weighted images and T_1_ mapping acquired at different time points post‐injection (0, 30, 90, 240, and 360 min).

As shown in Figure 3a, the T_1_‐weighted images of the USIO@Tmab group did not show significant contrast enhancement at 30 min. However, the tumor sites of mice showed progressively enhanced T_1_ contrast in MRI images with time and peaked at 240 min (Figure 3a), which was attributed to the enhanced permeability and retention (EPR) effect. This enhancement then diminished after 360 min. Quantitative results (Figure 3b) showed that the R_1_ of the tumors changed less at 30 min and peaked at 240 min with an approximate increase of 71.8%.

**FIGURE 3:**
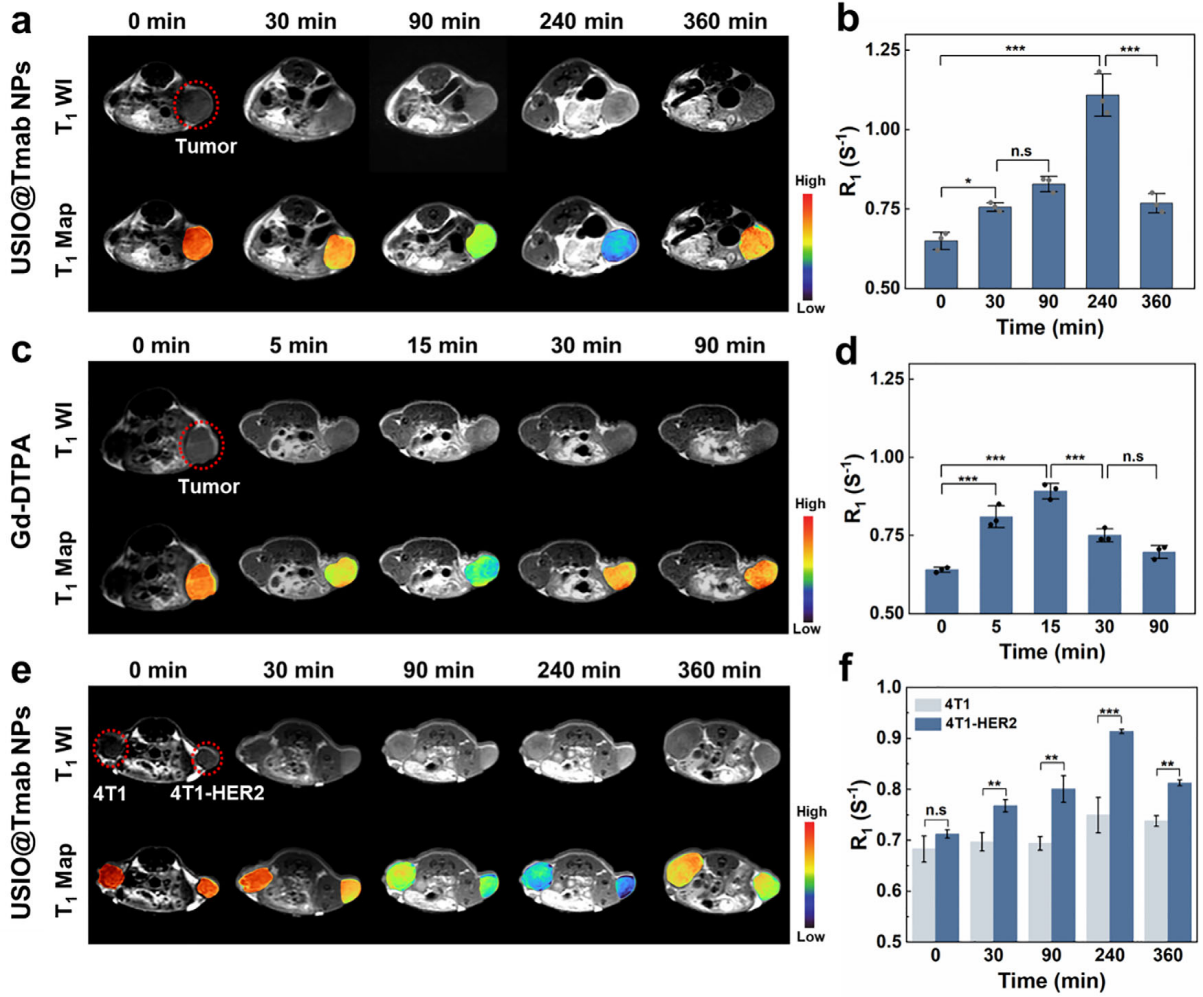
In vivo MR imaging performance of USIO@Tmab NPs in HER2‐positive breast tumor models. (a, c) Representative color‐coded T_1_ mapping images of the HER2‐positive tumor treated with either USIO@Tmab NPs (a) or Gd‐DTPA (c) at different time points. (b, d) Graph illustrating the R_1_ values in the tumor site at different time points of USIO@Tmab NPs (b) and Gd‐DTPA (d). (e) USIO@Tmab for in vivo MRI of 4T1 (left side) and 4T1‐HER2 (right side) xenograft tumor‐bearing mice. (f) R_1_ at both tumor sites in the mice. T_1_ Maps are representative data from three mouse samples. Data are presented as mean ± SD (*n* = 3). n.s, not significant, ^*^ *p* < 0.05, ^**^ *p* < 0.01, and ^***^ *p* < 0.001.

In comparison, Gd‐DTPA showed no time‐dependent signal enhancement and was rapidly metabolized in vivo (Figure 3c). The T_1_‐weighted images of the Gd‐DTPA group peaked at 15 min and began to gradually weaken at this time. The peak R_1_ of the USIO@Tmab group was 25% higher than that of the Gd‐DTPA group (*p* = 0.006 < 0.05), which shows that the imaging effect of the USIO@Tmab group was superior to that of Gd‐DTPA (Figure 3d). These results highlight the impressive pH‐responsive T_1_ contrast enhancement properties of USIO@Tmab NPs, which are particularly advantageous for tumor‐specific imaging.

Based on the good in vivo pH‐responsive T_1_ contrast enhancement by USIO@Tmab NPs, we further investigated the targeting of USIO@Tmab NPs as an in vivo MRI contrast agent. Consistent with the imaging time of unilateral tumors, after injection of USIO@Tmab NPs, the T_1_ contrast of the mouse tumor site in MRI images was progressively enhanced over time and peaked at 240 min (Figure 3e). At the same time point after injection, the right 4T1‐HER2 tumor sites were all significantly brighter than the left 4T1 tumors (red‐circled portion in Figure 3e). This can also be verified by analyzing the R_1_ data (Figure 3f), where the gap between the R_1_ values of the 4T1‐HER2 tumor‐forming cells and those of the 4T1 tumor‐forming cells gradually widened from 0 min to 240 min, with the R_1_ values of the 4T1‐HER2 tumors being 23% higher than those of the 4T1 tumors at the peak 240 min. These results suggest that USIO@Tmab NPs are highly promising for MRI forward imaging against HER2 breast cancer.

To further investigate whether the enhanced permeability and retention (EPR) effect contributes to tumor accumulation of USIO@Tmab NPs, we additionally evaluated a non‐pH‐responsive control (USIO NPs) in vivo. As shown in Figure S9, free USIO NPs produced only moderate T_1_ signal enhancement after intravenous administration, with the tumor R_1_ value increasing from 0.76 to 1.13 s^−1^ at 60 min postinjection before gradually returning toward baseline. In contrast, USIO@Tmab generated significantly stronger MRI enhancement in HER2‐positive tumors. These findings indicate that passive accumulation mechanisms, including the EPR effect, may contribute to tumor localization of the nanoparticles. However, they cannot fully account for the enhanced MRI signals observed with USIO@Tmab NPs. The superior imaging performance of USIO@Tmab NPs is likely governed by multiple factors, including EPR‐mediated passive accumulation, HER2‐mediated tumor retention, acidic TME‐triggered T_1_ activation, and liver/spleen‐associated clearance.

We acknowledge that an orthotopic tumor model would more closely replicate the clinical presentation of HER2‐positive breast cancer. Nevertheless, the subcutaneous model was chosen in the present study because it enables precise tumor boundary delineation for MR imaging and quantitative R_1_ mapping, free from signal contamination from adjacent mammary gland or adipose tissue. This technical advantage allowed us to obtain robust imaging metrics with minimal anatomical interference. It should also be noted that the in vivo MRI experiments were conducted with a relatively limited sample size (*n* = 3 per group). Although consistent imaging trends were observed across all animals, we recognize that larger cohorts would further enhance the statistical power and generalizability of our findings. Future studies using orthotopic models with expanded sample sizes are warranted to validate the translational potential of the USIO@Tmab nanoprobe.

### 2.6 Tumor Segmentation and Visualization via 3D nnU‐Net

To improve tumor visualization and support tumor assessment, MRI volumes acquired from mice injected with USIO@Tmab NPs or Gd‐DTPA were processed using a 3D nnU‐Net framework (Figure 4). The 3D architecture directly analyzes volumetric MRI data and incorporates spatial information across adjacent slices. The resulting segmentation masks were overlaid on the corresponding MR images to highlight tumor regions.

**FIGURE 4:**
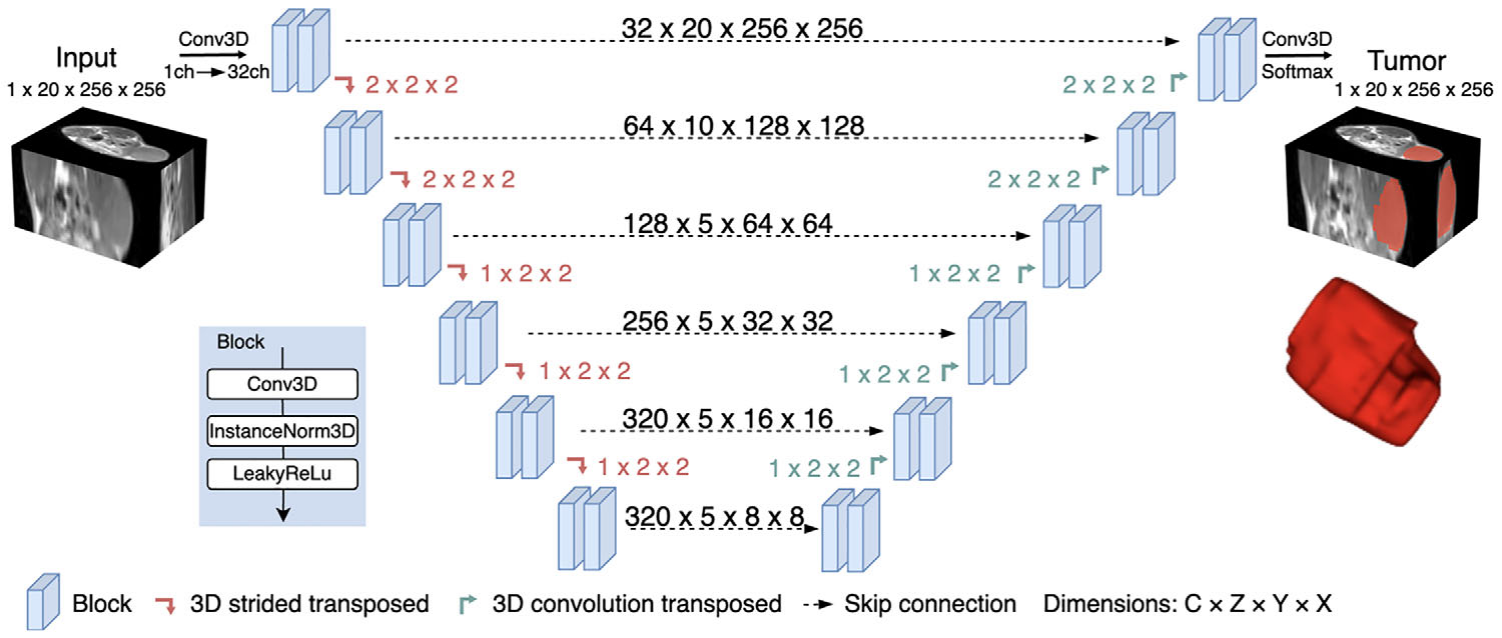
Network architecture of the 3D nnU‐Net used for volumetric tumor segmentation.

Figure 5 presents the segmentation results for six mice included in the comparative imaging experiments, including three mice injected with USIO@Tmab NPs and three injected with Gd‐DTPA. The segmentation result for each mouse was obtained from the test set of its corresponding fold in the case‐level five‐fold cross‐validation. The first row shows the contrast agent‐enhanced MR images, while the second and third rows show the ground truth (manual annotations) and 3D nnU‐Net predictions, respectively. The predicted tumor boundaries were generally consistent with the manual annotations, indicating that the model can automatically delineate tumors from volumetric MRI data.

**FIGURE 5:**
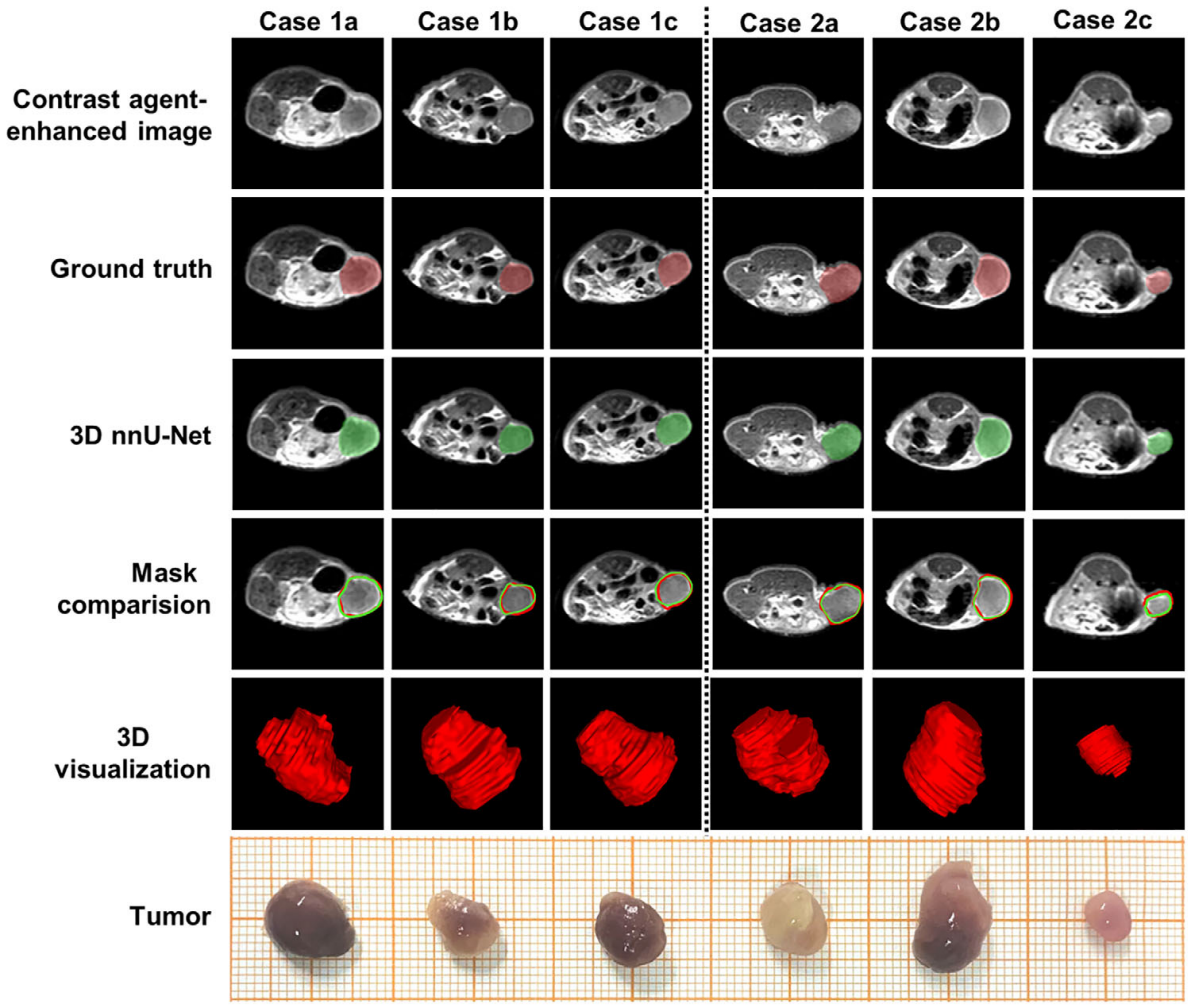
Segmentation and visualization results for two representative mouse groups (case 1: USIO@Tmab NPs; case 2: Gd‐DTPA) at the time point corresponding to the peak tumor R_1_. For each mouse, the segmentation result was obtained from the test set of its corresponding fold in the case‐level five‐fold cross‐validation. Shown are the contrast‐agent‐enhanced MR images, ground truth (manual annotations), 3D nnU‐Net predictions, mask comparisons, 3D tumor visualizations rendered in Python, and photographs of isolated paraformaldehyde‐fixed tumors.

Across the complete five‐fold cross‐validation on all mice, the 3D nnU‐Net achieved a Precision of 0.9365 ± 0.0316, Recall of 0.9322 ± 0.0322, Dice coefficient of 0.9336 ± 0.0201, and IoU of 0.8762 ± 0.0346. For the USIO@Tmab and Gd‐DTPA groups, the Dice coefficients were 0.9341 ± 0.0241 and 0.9227 ± 0.0593, respectively (Table S1). These results demonstrate stable tumor segmentation performance across the five cross‐validation folds and the two imaging groups.

The fifth row of Figure 5 shows 3D tumor visualizations generated from the 3D nnU‐Net‐predicted segmentation masks, while the sixth row presents the corresponding excised tumors for comparison. The visualized tumor shapes were generally consistent with the gross morphology of the excised tumors, indicating that the predicted masks preserved the main structural characteristics of the tumors. These 3D visualizations provide a more intuitive representation of tumor morphology and spatial extent and may support further assessment of tumor structure.

Additionally, tumor‐to‐normal ratios (TNR) for mice injected with USIO@Tmab contrast agents were quantified from contrast agent‐enhanced T_1_‐weighted images and 3D nnU‐Net‐processed images (Figure 6). The TNR value increased from 2.72 in the nanoprobe‐enhanced image to 7.07 after 3D nnU‐Net processing, corresponding to an approximate 2.59‐fold improvement in tumor‐to‐normal contrast relative to the unprocessed T_1_‐weighted images (Figure 6b). This phenomenon also occurred when a commercial Gd agent (Gd‐DTPA) was used (Figure 6c). Notably, USIO@Tmab NPs exhibited a higher TNR value than Gd‐DTPA in the unprocessed images. Importantly, we have made a clear distinction between the intrinsic contrast enhancement conferred by the USIO@Tmab nanoprobe (physical T_1_ shortening) and the computational visualization support offered by 3D nnU‐Net (segmentation overlay). The latter improves the visual tumor‐to‐normal contrast in fused images, thereby aiding intraoperative interpretation without altering the underlying MR signal. In summary, our experimental results demonstrate that USIO@Tmab NPs enable effective in vivo visualization of tumor sites. Moreover, the developed deep learning network can clearly delineate tumor boundaries and further enhance the contrast between tumors and surrounding tissues.

**FIGURE 6:**
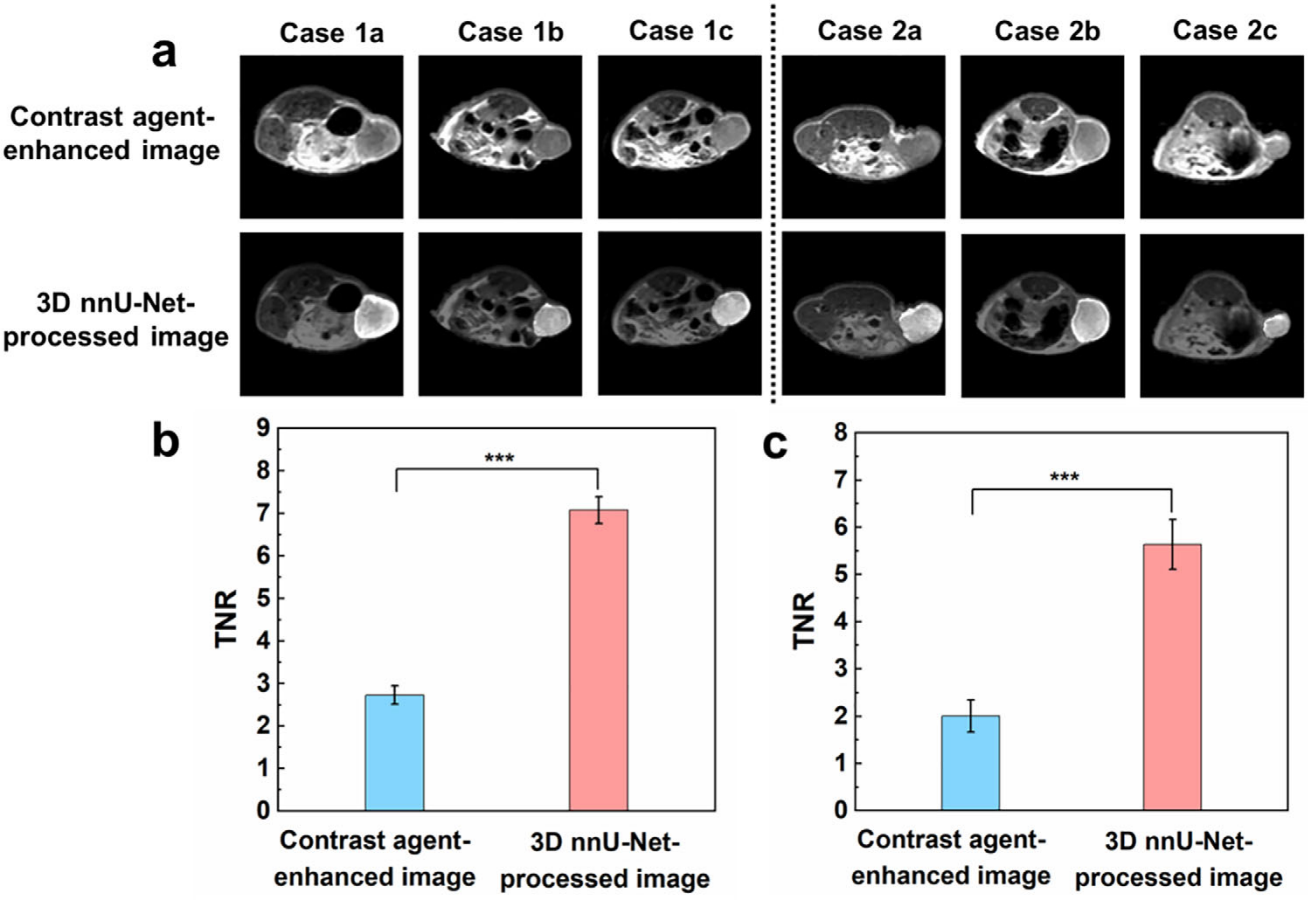
(a) T_1_‐weighted MR images of tumors acquired with contrast agent enhancement and subsequently processed by 3D nnU‐Net framework (case 1: USIO@Tmab NPs; case 2: Gd‐DTPA). (b, c) TNR values obtained from contrast agent‐enhanced and 3D nnU‐Net‐processed images of tumors in mice after injection of USIO@Tmab NPs (b) or Gd‐DTPA (c). Data are presented as mean ± SD (*n* = 3), and ^***^ *p* < 0.001.

### 2.7 Pharmacokinetics and Histology Toxicity Analysis

To assess pharmacokinetics of USIO@Tmab NP in vivo, blood iron concentrations were measured at different time points following intravenous administration. As shown in Figure S10, USIO@Tmab exhibited a blood circulation half‐life of approximately 98 min. The blood concentration gradually decreased over time while maintaining sufficient systemic exposure during the early post‐injection period, which is consistent with the observed tumor imaging performance. The relatively prolonged circulation time is expected to facilitate nanoparticle delivery to tumor tissues and provides a reasonable basis for the enhanced MRI signals observed in vivo.

To further evaluate the biosafety profile, the in vivo toxicity of USIO@Tmab NPs was systematically investigated. After 30 days of administration at an iron dose of 3 mg/mL (100 µL), major organs from the treated mice were harvested, sectioned, and subjected to hematoxylin and eosin (H&E) staining (Figure S11). Histopathological examination revealed no significant signs of tissue damage, such as necrosis or inflammatory infiltration, in any of the examined organs compared to the PBS control group. To evaluate the blood compatibility of USIO@Tmab NPs, a hemolysis assay was conducted (Figure S12). The USIO@Tmab NPs caused only minimal hemolysis, with rates remaining below 10% at all tested concentrations, whereas the positive control (Triton X‐100) resulted in almost complete hemolysis. These findings suggest that USIO@Tmab has favorable hemocompatibility for intravenous administration. Collectively, these results demonstrate that USIO@Tmab NPs possess satisfactory organ biocompatibility and a favorable in vivo safety profile.

## 3 Conclusions

In summary, this study presents a pH‐responsive and HER2‐targeted MRI contrast agent based on trastuzumab‐conjugated ultrasmall iron oxide nanoparticles (USIO@Tmab NPs), integrated with an advanced deep learning framework (3D nnU‐Net) to achieve highly sensitive and precise tumor delineation. The combined strategy delivers a 2.72‐fold TNR enhancement via nanoprobe‐mediated T_1_ shortening, complemented by a 2.59‐fold improvement in tumor boundary visualization through AI‐assisted segmentation, without any physical signal amplification from the deep learning model. The work provides a mechanistic insight into tumor‐microenvironment‐responsive contrast enhancement and establishes a robust platform for accurate diagnosis and surgical guidance in HER2‐positive breast cancer. Collectively, our approach demonstrates considerable potential for clinical translation toward more effective and individualized cancer management.

## 4 Experimental Section

### 4.1 Materials

FeCl_3_·6H_2_O, diethylene glycol (DEG), fluorescein isothiocyanate (FITC), and ammonium chloride (NH_4_Cl) were obtained from Macklin (Shanghai, China). L‐ascorbic acid and sodium acetate were obtained from J&K Scientific (Beijing, China). Ethanol, anhydrous ethanol, and dimethyl sulfoxide were provided by Aladdin (Shanghai, China). Sodium citrate (Na_3_Cit) was acquired from Accela (Shanghai, China). Tmab antibody was purchased from Roche (Shanghai, China). Cell counting kit‐8 (CCK‐8) assay kit, 4’,6‐diamidino‐2‐phenylindole (DAPI), and 4% paraformaldehyde were obtained from Beyotime (Shanghai, China). Dulbecco's modified Eagle medium (DMEM), phosphate‐buffered saline (PBS), bicinchoninic acid (BCA) assay kit, and 0.25% trypsin‐EDTA were purchased from Thermo Fisher Scientific (Waltham, MA).

### 4.2 Synthesis of USIO@Tmab NPs

The monodispersed USIO NPs were synthesized through the solvothermal method [54]. Specifically, 1.09 g of FeCl_3_·6H_2_O was dissolved in 40 mL of diethylene glycol. Then, 0.47 g of Na_3_Cit was dissolved in the above solution and vigorously stirred at 80°C under an air atmosphere for 1 h. After the Na_3_Cit was completely dissolved, 1.312 g of anhydrous sodium acetate was added to the above solution and stirred continuously until the sodium acetate powder was completely dissolved and the solution was transferred to a 50 mL high‐pressure reactor and reacted at 200°C for 4 h. The solution was then cooled to room temperature naturally. The formed USIO NPs were collected by magnetic decantation, washed with anhydrous ethanol twice, and dried in a vacuum oven at 60°C for 12 h. Tmab protein was used to coat USIO NPs through electrostatic adsorption. 1 mg of USIO NPs was added to the Tmab solution (3 mL, 1 mg/mL). The mixture was stirred for 30 min, and the remaining Tmab was removed by magnetic decantation. The USIO@Tmab NPs were resuspended in PBS and stored at 4°C for further use.

### 4.3 MRI Relaxivity and DWI of USIO@Tmab NPs

The T_1_ relaxivity of USIO@Tmab NPs was evaluated before and after exposure to acidic solutions at pH 7.4 and 5.0 using a Philips Ingenia 3.0 T MRI scanner equipped with an eight‐channel phased array wrist coil. The Fe^3+^ concentrations of USIO@Tmab NPs ranged from 0.1 to 0.6 mM under varying pH conditions. The specific parameters were set as follows: T_1_WI: TR = 500 ms, TE = 11.5 ms, slice thickness = 2 mm, slice spacing = 0.2 mm, field of view (FOV) = 144 mm × 144 mm; T_1_ map: TR = 4.2 ms, TE = 2.1 ms, slice thickness = 2 mm, slice spacing = 0 mm, FOV = 144 mm × 90 mm. The r_1_ (mM^−1^s^−1^) values were determined based on the relationship between the inverse T_1_ relaxation times (1/T_1_) and varying Fe^3+^ concentrations.

To elucidate the mechanism underlying the release of Fe_3_O_4_ nanoparticles from USIO@Tmab NPs due to protein disruption in acidic environments, USIO@Tmab NPs were incubated at a concentration of 0.05 mM in both acidic and neutral solutions before MR‐DWI scanning. The acquisition parameters were as follows. DWI: TR/TE = 3000 ms/76 ms, slice thickness = 1 mm, slice spacing = 0 mm, FOV = 144 mm × 144 mm. Images with two different b values (0 and 1000 s mm^−2^) were obtained, and the apparent diffusion coefficient (ADC) values were recorded.

### 4.4 In Vitro Cytotoxicity and Targeting

4T1 (RRID: CVCL_0125) and SKBR3 (RRID: CVCL_0033) cell lines were sourced from Procell Life Science & Technology Co., Ltd. (Wuhan, China). HER2 stable expressing 4T1 cells (4T1‐HER2) were acquired through plasmid and lentivirus infection [55]. 4T1‐HER2 and 4T1 cells were seeded into 96‐well plates at a density of 8000 cells per well. After overnight incubation, the cells were cocultured with USIO@Tmab at different concentrations for 24 h. The cell viabilities were measured using the CCK‐8 assay according to the manufacturer's instructions. The morphology of these treated cells was observed by a Leica DM IL LED fluorescence microscope (Wetzlar, Germany). The cell nuclei and cytoskeleton were stained with DAPI and phalloidin, respectively.

To evaluate in vitro specific targeting, SKBR3 cells (a HER2‐positive cell line) and 4T1 cells were inoculated into 12‐well plates at a density of 2 × 10^5^ cells per well, and cultured overnight. The cells were then cocultured with FITC‐modified USIO@Tmab for 6 h. Afterward, the cells were washed twice with PBS and fixed using 4% paraformaldehyde. The nucleus was then stained with DAPI before imaging by a Leica DM IL LED fluorescence microscope (Wetzlar, Germany).

### 4.5 In Vitro MR Properties

To assess pH responsiveness and HER2‐mediated targeting in vitro, we selected triple‐negative breast cancer cells (4T1, with acidic environment and without HER2 target), an HER2‐stable‐expressing cell model (4T1‐HER2, with acidic environment and HER2 target), and human normal mammary epithelial cells (MCF‐10A, with a neutral environment). To assess pH responsiveness and HER2‐targeting specificity in vitro, we employed a panel of three cell lines: HER2‐negative 4T1 cells (acidic environment), HER2‐positive 4T1‐HER2 cells (acidic environment), and normal MCF‐10A mammary epithelial cells (neutral environment). This design enabled independent evaluation of pH‐triggered behavior and HER2‐mediated recognition. Specifically, 4T1, 4T1‐HER2, and MCF‐10A cells were incubated with USIO@Tmab NPs (50 µg Fe/mL) for 2 h, washed three times with PBS, and then collected at designated time intervals. The harvested cells were fixed in 1.0% agarose within tubes and subjected to MR imaging on a 3.0 T clinical scanner (Ingenia, Philips). T_1_ mapping was performed with the following parameters: TR = 4.2 ms, TE = 2.1 ms, slice thickness = 2 mm, slice spacing = 0 mm, and FOV = 144 mm × 90 mm. T_1_ values were subsequently quantified from the reconstructed T_1_ maps using the 3.0 T ImageView workstation (Ingenia, Philips).

### 4.6 In Vivo MRI of Tumors

All the animal experiments were strictly performed and approved by the Animal Ethics Committee of the Shanghai General Hospital affiliated with Shanghai Jiaotong University (2024AW069). To establish the subcutaneous tumor models, 100 µL cell suspensions containing 1 × 10^6^ of 4T1‐HER2 cells were injected subcutaneously into the right back of the Balb/c nude mice. Animals with body weights of approximately 20 g and tumor volumes of about 100 mm^3^ (calculated as 1/2 × length × width^2^) were used for in vivo MR experiments. Mice were scanned on a 3.0 T MR scanner with a high‐resolution animal coil before and after injection of 200 µL USIO@Tmab (3 mg/mL of Fe) or commercial gadopentetic acid (Gd‐DTPA, 5 mg/mL of Gd). All mice were imaged under the T_1_WI spin‐echo sequence and T_1_ mapping sequence. For T_1_‐weighted images (WI), the parameters were TR = 550 ms, TE = 11.5 ms, slice thickness = 1 mm, slice spacing = 0 mm, FOV = 130 mm × 130 mm. For T_1_ mapping images, a Modified Look‐Locker Inversion Recovery (MOLLI) sequence was used [56]. A 5s(3s)3s scheme and a fixed simulated heart rate of 60 bpm (beats per minute) were used, which resulted in the following inversion times (ITs): 117, 350, 1117, 1350, 2317, 2550, 3317, 4317 ms. The other parameters of this T_1_ mapping sequence were flip angle (FA) = 20°, TR/TE = 5.8/2.9 ms, slice thickness = 1 mm, slice spacing = 0 mm and voxel size = 1.2 mm × 1.15 mm. The mean T_1_‐weighted signal intensities and T_1_ relaxation time were measured from each tumor. In vivo MR was further performed to verify the in vivo HER2 targeting property of USIO@Tmab. 4T1‐HER2 and 4T1 cells were respectively injected subcutaneously into the right and left back of the Balb/c nude mice to establish bilateral tumor‐bearing mice models. After the body weights and tumor volumes of the mice reached approximately 20 g and 100 mm^3^, the mice were intravenously injected with USIO@Tmab NPs for targeted MR imaging in vivo. The T_1_WI and T_1_ mapping images were obtained using the approach described above. During the experimental period, mice were monitored regularly for body weight and tumor growth. Predefined humane endpoints included substantial body weight loss (>20%), excessive tumor burden (>2000 mm^3^), and impaired mobility or feeding. Animals meeting any of these criteria were humanely euthanized.

### 4.7 Tumor Segmentation and Visualization Based on 3D nnU‐Net Architecture

To improve tumor visualization and support tumor assessment, we processed images through Python 3.9.0 acquired with the USIO@Tmab NPs contrast agent using a 3D nnU‐Net (nnU‐Net v2.5.2)‐based deep learning framework. The proposed 3D nnU‐Net‐based deep learning framework first segmented the tumor with 3D nnU‐Net and then enhanced image contrast by fusing the original MR images with the segmented tumor masks using Matplotlib 3.8.4. Meanwhile, in each case, we reconstructed the segmented tumor masks into three‐dimensional (3D) models via 3D Slicer 5.6.2, thereby offering clinicians an intuitive depiction of the tumor's morphological features.

The proposed 3D nnU‐Net‐based framework comprised five main steps: preprocessing, network configuration and training, tumor mask prediction, and tumor mask postprocessing. Initially, the preprocessing step normalized input MRI images to ensure reliable subsequent network training. Specifically, the input volume was resized to 20 × 256 × 256, and z‐score normalization was performed by subtracting the mean and dividing by the standard deviation.

We configured a deep learning network based on 3D nnU‐Net, which demonstrated superior performance in biomedical image segmentation tasks, particularly for tumor segmentation. Key hyperparameters of the 3D nnU‐Net were optimized to enhance tumor segmentation performance. The constructed network used a full‐resolution 3D U‐shaped architecture, with a contracting path (encoder) for multi‐resolution feature extraction from input MRI images and a symmetric expanding path (decoder) for tumor region prediction. A six‐level structure was adopted, incorporating six downsampling operations. Skip connections bridge the encoder and decoder by transferring feature maps from each encoder level to the corresponding decoder level. This design facilitates gradient flow during backpropagation, improving training efficiency. The network was trained using MRI volumes from the training set, with corresponding manually segmented tumor masks as target outputs. After training, the trained 3D nnU‐Net generates a tumor likelihood map for any unseen MRI volume.

The imaging dataset comprised MRI data from 10 murine models, from which a total of 1,827 annotated MRI slices were obtained. Model performance was evaluated using case‐level five‐fold cross‐validation. In each fold, two mice were used as the test set, while the remaining eight mice were used for model training and validation. All slices and the complete MRI volume from each mouse were assigned to the same subset to prevent data leakage. Across the five folds, each mouse served once as a test case. Hyperparameters (e.g., learning rate, batch size) were optimized using the validation data within each fold. The 3D nnU‐Net was trained using a combined Dice and binary cross‐entropy loss, and its performance was evaluated on the corresponding test cases using precision, recall, Dice coefficient, and intersection over union (IoU). The overall performance was reported as the mean and standard deviation across the five folds. The final tumor segmentation masks were obtained by binarizing the 3D nnU‐Net‐generated probability map at a threshold of 0.5. For visualization, each predicted mask was overlaid on the corresponding MR image using weighted blending (25% mask, 75% grayscale), highlighting tumor regions while preserving anatomical context. Furthermore, for each mouse, the predicted volumetric segmentation mask was further used to generate a 3D tumor visualization, providing an intuitive representation of tumor morphology and spatial extent.

### 4.8 Statistical Analysis

Data are expressed as mean ± standard deviation (SD). An unpaired two‐tailed Student's t‐test was used for comparisons between two groups, whereas a one‐way ANOVA was applied for multi‐group comparisons (involving three or more groups). Statistical significance was set at ^*^ *p *< 0.05, ^**^ *p *< 0.01, and ^***^ *p *< 0.001; n.s denotes not significant.

## Author Contributions

**Yi Zhu**: methodology, writing – original draft, data curation, software. **Xiangyuan Ma**: supervision, writing – review and editing, methodology, software. **Jiaying Zheng**: methodology, writing – original draft, data curation, software. **Sunxian Dai**: data curation, software. **Xinrui Li**: data curation. **Hongwei Lu**: supervision, writing – review and editing. **Zhongling Wang**: resources, writing – review and editing, funding acquisition, supervision. **Benqing Zhou**: conceptualization, funding acquisition, project administration, resources, writing – review and editing, supervision.

## Conflicts of Interest

The authors declare no conflicts of interest.

## Supporting information




**Supporting File**: advs77355‐sup‐0001‐SuppMat.docx.

## Data Availability

The data that support the findings of this study are available on request from the corresponding author. The data are not publicly available due to privacy or ethical restrictions.
