# Supplementary material for: AI‐Assisted Tumor Boundary Delineation via Targeted Ultrasmall Iron Oxide Nanoprobe for High‐Contrast HER2‐Positive Tumor Imaging
Source: Adv Sci (Weinh). 2026 Aug 24;13(63):e77355. doi: 10.1002/advs.77355 (PMC13502355; doi:10.1002/advs.77355)
Supplement: Supplementary file 1 [file ADVS-13-e77355-s001.docx]

**Supporting Information**

**AI-Assisted Tumor Boundary Delineation via Targeted Ultrasmall Iron Oxide Nanoprobe for High-Contrast HER2-Positive Tumor Imaging**

Jiaying Zheng^a, 1^, Yi Zhu^b, 1^, Xinrui Li^a^, Sunxian Dai^b^, Xiangyuan Ma^a,^ *, Hongwei Lu^a,^ *, Zhongling Wang^b,^ *, Benqing Zhou^a,^ *

^a^ Department of Biomedical Engineering, College of Engineering, Shantou University, Shantou, 515063, PR China

^b^ Department of Radiology, Shanghai General Hospital, Shanghai Jiaotong University School of Medicine, Shanghai, 200080, PR China

^____________________________________________________________________________^

* Corresponding author. E-mail address: maxiangyuan@stu.edu.cn (X. Ma), [hwlu@stu.edu.cn](mailto:hwlu@stu.edu.cn) (H. Lu), [zlwang138136@126.com](mailto:zlwang138136@126.com) (Z. Wang), bqzhou@stu.edu.cn (B. Zhou).

^#^ Authors contributed equally to this work.

Table S1. Segmentation performance of the 3D nnU-Net based on case-level five-fold cross-validation. The overall results are presented as mean ± SD across the five folds, while the group-specific results are calculated from the corresponding cross-validation test cases.

| **Evaluation set** | **Precision** | **Recall** | **Dice** | **IoU** |
| --- | --- | --- | --- | --- |
| Overall five-fold cross-validation | 0.9365 ± 0.0316 | 0.9322 ± 0.0322 | 0.9336 ± 0.0201 | 0.8762 ± 0.0346 |
| USIO@Tmab NPs | 0.9442 ± 0.0304 | 0.9256 ± 0.0396 | 0.9341 ± 0.0241 | 0.8773 ± 0.0421 |
| Gd-DTPA | 0.9209 ± 0.0829 | 0.9304 ± 0.0316 | 0.9227 ± 0.0593 | 0.8607 ± 0.0764 |

Figure S1. A TEM image (a), TEM size distribution histogram (b), and hydrodynamic size distribution histogram (c) of USIO NPs.

Figure S2. X-ray diffraction patterns (a) and electron diffraction pattern of selected areas (b) of the USIO NPs.

Figure S3. Full XPS spectra of the USIO NPs (a), XPS spectra (700-740) of high-resolution Fe2p (b), and XPS spectra (280-290) of high-resolution C1s (c).

Figure S4. TEM size distribution histogram (a), and hydrodynamic size distribution histogram (b) of USIO@Tmab.

Figure S5. The standard curve for BSA protein in the BCA protein assay.

Figure S6. Hydrodynamic size of the USIO and USIO@Tmab NPs dispersed in water at different time periods. Photographs of the USIO (left) and USIO@Tmab NPs (right) dispersed in cell culture medium with 10% FBS (1), water (2), and PBS (3), respectively for 48 hours. Data are shown as mean ± SD (n = 3).

Figure S7. T_1_-weighted color-coded images and linear fitting of 1/T_1_ as a function of Fe concentration of USIO NPs at different Fe concentrations.

Figure S8. Microscope images of 4T1-HER2 (a) and 4T1 cells (b) cocultured with USIO@Tmab NPs at different concentrations for 24 h.

Figure S9. (a) Representative T_1_-weighted and T_1_ map images of tumor-bearing mice with injection of USIO NPs. (b) R1 values in the tumor site at different time points. Red circle refers to the tumor site. Data are presented as mean ± SD (n = 3), and ***p < 0.001.

Figure S10. In vivo blood pharmacokinetics of USIO@Tmab NPs after intravenous injection in mice. Blood samples were collected at various time points post-injection, and the iron concentration was measured. Data are shown as mean ± SD (n = 3).

Figure S11. Histological changes in the heart, kidney, liver, lung, and spleen of the mice at 1 month post intravenous injection of the USIO@Tmab NPs ([Fe] = 3 mg/mL, 100 μL). Mice injected with 100 μL PBS were used as control.

Figure S12. Hemolysis test of USIO@Tmab NPs at different concentrations (0–50 μg/mL). Inset: photographs of hemolysis with concentrations increasing from left to right (0, 0.1, 1, 2, 5, 10, 50 μg/mL), with Triton X-100 as the positive control (far right).
